# An economic analysis of BPaL for multidrug-resistant TB in South Africa and the Philippines

**DOI:** 10.5588/ijtldopen.25.0294

**Published:** 2025-09-10

**Authors:** S.D. Masuku, C. Nattey, L. Coetzee, K. Hirasen, A. Mabhula, D.J. Casalme, M.T. Gler, A. Gupta, S. Juneja, N. Ndjeka, D. Evans, B.E. Nichols

**Affiliations:** ^1^ Health Economics and Epidemiology Research Office, Faculty of Health Sciences, University of the Witwatersrand, Johannesburg, South Africa;; ^2^ SCHARR, Sheffield Centre for Health and Related Research, Division of Population Health, School of Medicine and Population Health, University of Sheffield, Sheffield, UK;; ^3^ De La Salle Health Sciences Institute, Cavite, Philippines;; ^4^ TB HIV Innovations and Clinical Research Foundation, Cavite, Philippines;; ^5^ TB Alliance, New York, NY, USA;; ^6^ TB Control and Management, National Department of Health, Pretoria, South Africa;; ^7^ Department of Medicine, University of Cape Town, Cape Town, South Africa;; ^8^ Department of Global Health, Amsterdam Institute for Global Health and Development, Amsterdam UMC, University of Amsterdam, Amsterdam, The Netherlands;; ^9^ Department of Global Health, Boston University School of Public Health, Boston, MA, USA;; ^10^ Wits Diagnostic Innovation Hub, Faculty of Health Sciences, University of the Witwatersrand, Johannesburg, South Africa.

**Keywords:** tuberculosis, MDR-TB, bedaquiline, budget impact, economic impact, BPaL

## Abstract

**BACKGROUND:**

The WHO endorses bedaquiline, pretomanid, and linezolid (BPaL)-based regimens for multidrug-resistant/rifampicin-resistant TB, and both the Philippines (PH) and South Africa (SA) have adopted these regimens.

**METHODS:**

Using a Markov model, we assessed the cost per successful treatment and 5-year budgetary and economic impact of BPaL-based regimens in SA and PH. Treatment outcomes were informed by national electronic registries, SA BPaL Clinical Access Program, and PH operational research. Costs were estimated from the provider perspective.

**RESULTS:**

Over 5 years, BPaL-based regimens reduce total costs by 22%–28% in SA and 13%–16% in PH compared with a standard short oral regimen (SSOR) when achieving the same number of successful treatments, due to lower cost per successful treatment from reduced loss to follow-up and mortality. BPaL-based regimens improve treatment success by 26%, leading to more patients completing full treatment and higher overall resource use. Therefore, the budget for BPaL-based regimens is projected to increase by 7%–8% (SA) and 6% (PH) from 2023/24 to 2027/28.

**CONCLUSION:**

BPaL-based regimens reduce cost per successful treatment compared with SSOR and require smaller budgets for similar treatment outcomes. Implementation may involve initial budget increases, but improvements in treatment success and long-term health outcomes outweigh these costs, presenting a strong rationale for rollout.

In 2022, the WHO recommended the 24-week all-oral regimen of bedaquiline, pretomanid, and linezolid (BPaL), and bedaquiline, pretomanid, linezolid, and moxifloxacin (BPaLM) for treating multidrug-resistant and rifampicin-resistant TB (MDR/RR-TB), replacing longer standard regimens.^1^ The Philippines (PH) followed these guidelines,^1^ while South Africa (SA) implemented the bedaquiline, pretomanid, linezolid, and levofloxacin (BPaL-L) regimen, substituting moxifloxacin with levofloxacin.^2^ For fluoroquinolone-resistant (FQ-R) cases, two treatment options are available: BPaL, which is recommended,^1^ and an individualised 18-month regimen. Previous studies estimated that BPaL-based regimens would cost substantially less than the standard short oral regimen (SSOR) or standard long oral regimen (SLOR) in both SA^3^ and PH.^4^ BPaL for treating extensively drug-resistant TB could also result in cost savings.^5,6^ However, no study has estimated the economic and budgetary impact of implementing BPaLM/L as the standard of care for treating MDR/RR-TB (Supplementary Data Table S1).^7–10^

We built a Markov model to analyse the cost per treatment success, projected 5-year budgetary impact, cost per disability-adjusted life year (DALY) averted, and economic impact of introducing BPaL-L in SA or BPaLM in PH or BPaL for FQ-R cases (20% of MDR/RR-TB cases).^11–12^

## METHODS

We used country-specific national electronic registries to estimate the number of patients initiating treatment over 5 years. SA BPaL Clinical Access Program (BCAP) and PH operational research (OR) primary data informed transition probabilities and treatment outcomes for BPaL and SSOR.^4,5^ Guidelines informed quantities of drugs, diagnostics, and laboratory tests needed.

### Study population

For SA, we analysed the Electronic Drug-Resistant Tuberculosis Register (EDRWeb) for adults with MDR/RR-TB who began SSOR/SLOR treatment between 1 January 2019 and 31 December 2021, ensuring treatment outcomes were recorded by February 2023. For PH, we analysed the Integrated Tuberculosis Information System (ITIS) data extracted in June 2023 covering the same period (2019–2021).

To project 2024/25 treatment initiates in SA, we used 2018/19^13^–2022/23 average registrations, holding this constant through 2027/28 for simplicity. Data beyond 2022/23 were projections. Fiscal years (e.g., 2023/24) were used instead of calendar years to align with the government’s budgeting and planning cycles.

For PH, 2022/23 initiates were based on ITIS data, with a projected 22% increase for 2023/24 and 20% annually thereafter, informed by Programmatic Management of Drug-Resistant Tuberculosis (PMDT) projections for a new case-finding strategy (Table 1).

**Table 1.** Estimated number of patients initiating treatment for MDR/RR-TB and projected BPaL-based regimen: SSOR treatment initiation ratio by rollout strategy.

	Cohort				
	2023/24	2024/25	2025/26	2026/27	2027/28
Number of treatment initiations					
South Africa	8,483	9,085	9,085	9,085	9,085
Philippines	9,143	10,972	13,166	15,799	18,959
Rollout strategies (BPaL-based regimen: SSOR)					
Baseline	0:100	0:100	0:100	0:100	0:100
Strategy 1: aggressive rollout	80:20	90:10	90:10	90:10	90:10
Strategy 2: moderate rollout	80:20	80:20	85:15	90:10	90:10
Strategy 3: slow rollout	30:70	60:40	70:30	80:20	80:20

BPaL = short, all-oral, 24-week regimen comprising bedaquiline, pretomanid, and linezolid (600 mg), with or without moxifloxacin (M)/levofloxacin (L); SSOR = standard short oral regimen; SLOR = standard long oral regimen; MDR/RR-TB = multidrug- and rifampicin-resistant TB.

We compared three rollout strategies for the BPaL-based regimen – Aggressive, Moderate, and Slow – against baseline where all MDR/RR-TB patients receive SSOR (Table 1).

### Model design and assumptions

**Figure 1.**
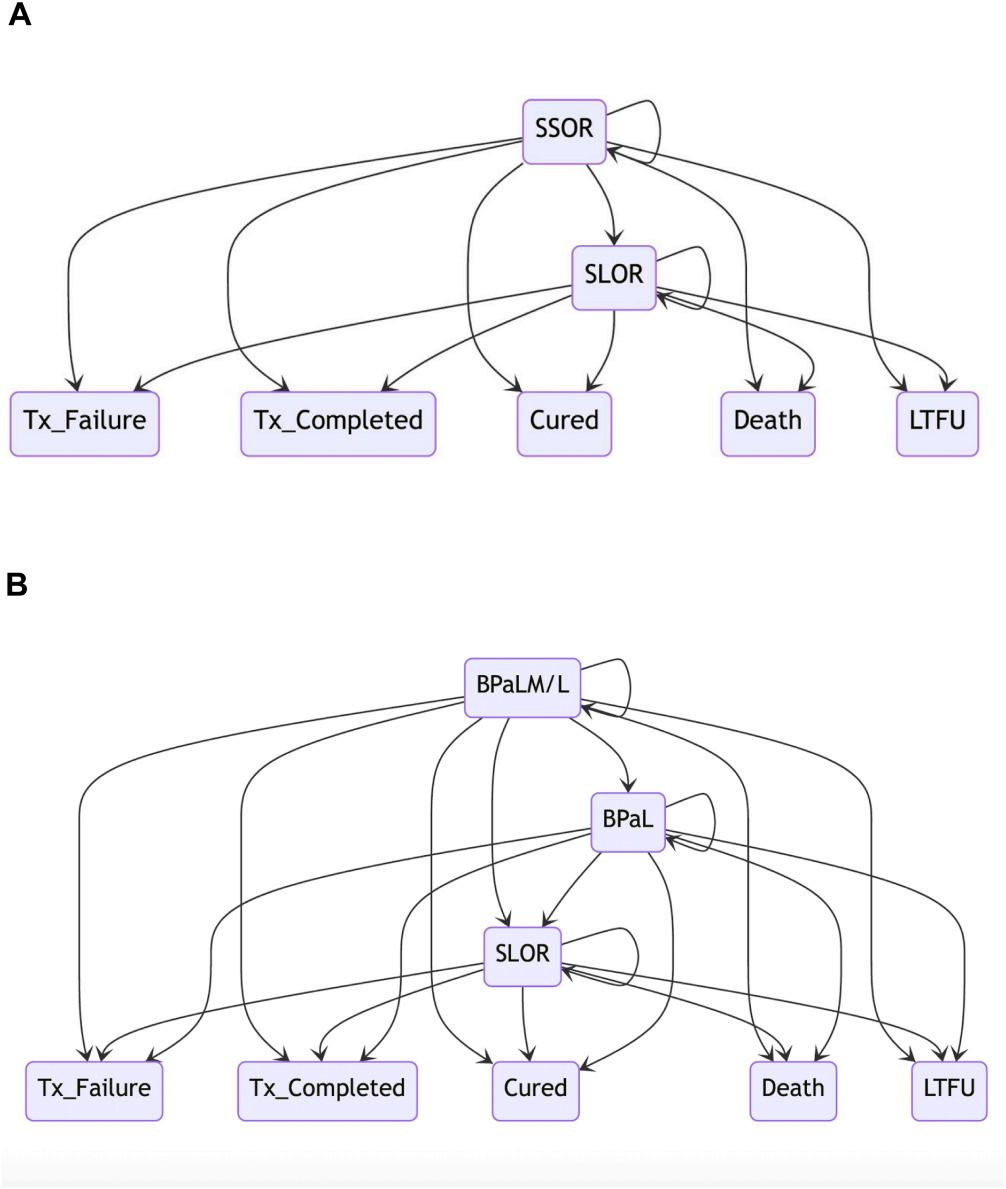
Structure of the Markov model and the relationship between different health states. Patients are assigned to **A:** SSOR or **B:** a BPaL-based regimen (BPaLM/L or BPaL), based on eligibility and country rollout strategy. BPaL = short, all-oral, 24-week regimen comprising bedaquiline (400 mg daily for 2 weeks followed by 200 mg three times per week for 22 weeks), pretomanid (200 mg daily), and linezolid (600 mg daily for 16 weeks, then 300 mg daily for 8 weeks), with or without 400 mg moxifloxacin (M) or levofloxacin (L). SSOR = standard short oral regimen. SLOR = standard long oral regimen; LTFU = loss to follow-up; Tx_Failure = treatment failure; Tx_Completed = treatment completed.

We developed a Markov model that tracked MDR/RR-TB patients through treatment stages and assessed costs and outcomes over five fiscal years (2023/24–2027/28) under different rollout strategies (Figure 1). The model followed MDR/RR-TB patients eligible for a 24-week BPaL-based regimen or a 9- to 11-month SSOR regimen from initiation to final outcome, with monthly transitions to the next treatment month or to loss to follow-up (LTFU), treatment failure (i.e., delayed or reversed culture conversion), death, or treatment success (cure or completion).^2^ Treatment outcomes – completed, cured, LTFU, failure, or death – were recorded using WHO standard case definitions.^14,15^ Patients were assumed to start treatment midyear, to reflect even initiation across the year and simplify time-based modelling. Patients intolerant of or failing a BPaL-based regimen or SSOR transition to a longer oral regimen (SLOR), incurring its associated costs.^16^

### Transition probabilities

Monthly probabilities of LTFU, treatment failure, and death were derived from national electronic registries for SSOR and SLOR and from the SA BCAP for BPaL and were applied to PH in the absence of local data. We assumed transition probabilities for BPaLM/L would be the same as BPaL.^4,5^ Details on the BCAP and transition probabilities are in Supplementary Data.^17–21^ Table 2 presents the proportions of patients in each health state, by regimen and country, at the scheduled end of treatment.

**Table 2.** Distribution of patients across treatment outcome categories, categorised by regimen and country.

	SSOR^A^		BPaLM/L	BPaL	SLOR^A^	
	South Africa	Philippines	South Africa^B^/Philippines	South Africa^B^/Philippines	South Africa	Philippines
Total mortality rate, %	16.88	8.26	2.56	2.56	20.10	22.93
Total LTFU rate, %	14.68	7.34	0.00	0.00	21.76	6.06
Total treatment success, %	66.97	83.24	97.44	97.44	53.46	66.48
Total treatment failure, %	1.47	1.16	0.00	0.00	4.40	4.53

BPaL = short, all-oral, 24-week regimen comprising bedaquiline, pretomanid, and linezolid (600 mg), with or without moxifloxacin (M)/levofloxacin (L); SSOR = standard short oral regimen; SLOR = standard long oral regimen; LTFU = loss to follow-up.

A From country-specific national electronic registries.

B From the South African BPaL Clinical Access Program (BCAP).

### Cost data

Visit costs (including overhead, equipment, and staffing) were obtained from costing studies conducted as part of the SA BCAP and PH OR (Supplementary Data). Further details on these costs can be found in the primary cost and cost-effectiveness publications.^4,5^ For this analysis, it was assumed that the duration of BPaL-based regimen visits would be comparable to those observed in the SA BCAP and PH OR studies. To replicate programmatic conditions, we used a guidelines approach to inform quantities of drugs, diagnostics, and laboratory tests needed for BPaLM/L, BPaL, SSOR, and SLOR. In SA, estimates were based on the 2023 updated clinical reference guide,^2^ whereas WHO guidelines were used for PH due to lack of current national guidance.^1^ Drug costs for SA were sourced from the National Department of Health’s master procurement catalogue, while pretomanid pricing was obtained from the Global Drug Facility (GDF) product catalogue.^22^ Laboratory test costs for SA were obtained from the National Health Laboratory Service’s 2018 state price list.^23^ For PH, unit costs for laboratory tests and procedures were sourced from costing data from the programme implementer in 2023. Where applicable, TB-specific and ancillary drug costs were sourced from Stop TB partnership’s GDF product catalogue.^4,14,24^

### Cost per patient successfully treated and economic impact of DALYs averted

We calculated the cost per patient successfully treated by dividing total cohort costs (for patients initiating treatment within the same year) by the number who successfully completed treatment. Monthly health state costs were estimated by multiplying resources used, unit costs, and patient numbers. Total costs were analysed by cohort and budget year. We also estimated the total cost to match baseline treatment successes using: cost per success under each strategy × baseline successes.

To estimate economic value, we multiplied DALYs averted over 5 years (2023–2027) for BPaL-based regimens vs. SSOR^7^ by the gross domestic product (GDP) per capita: US$6,022.50 for SA and US$3,804.90 for PH.^25^ This approach, previously used to estimate the monetary value of DALYs lost from disease,^26^ offers a consistent framework for comparing the economic impact of health interventions without assigning different economic values to health within a country,^25^ even if GDP per capita may not precisely represent the average economic gains of individuals affected by TB. When necessary, costs were adjusted for inflation using country-specific inflation rates.^27^ All costs were reported in 2023 US dollars, based on average exchange rates between 13 January 2023 and 13 October 2023 (US$1 = SA rand 18.38 and US$1 = PH peso 55.60). Costing was conducted from the perspectives of the respective National TB Control Programs.

### Sensitivity analysis

To evaluate the robustness of our model, we performed a one-way sensitivity analysis on the cost per patient successfully treated in 2027/28, focussing on Strategy 2 in SA (the most likely rollout). We varied key parameters: ±1.5% in cost inflation, BPaLM/L LTFU, treatment failure, and mortality rates set equal to SSOR, and ±20% in SLOR per-patient costs to capture over- or underestimation of treatment failure under BPaL-based regimens or SSOR.

### Ethical statement

The study protocol was approved by the Human Research Ethics Committee (Medical) of the University of the Witwatersrand, Johannesburg, SA (HREC protocol number M220140), and the Asian Hospital and Medical Center Research Ethics Committee, Alabang, Muntinlupa City, Philippines. The study used programmatic data, and a waiver of informed consent was granted to review country-specific national electronic registers retrospectively.

## RESULTS

BPaL-based regimens are projected to increase treatment success by 26% (range: 15%–45%) over 5 years compared with baseline (SSOR). They reduce rates of LTFU, treatment failure, and mortality but may increase average treatment duration, as fewer patients are lost to follow-up in the early months of treatment (e.g., LTFU drops from 1.47% in SA and 1.16% in PH for SSOR to 0% for BPaL-based regimens). Therefore, with nearly 97% of patients completing treatment on BPaL-based regimens – vs. 67% in SA and 83% in PH on SSOR – managing a cohort of MDR/RR-TB patients initiating treatment within the same year would require more resources, including medications, monitoring visits, and laboratory tests.

The cost per successful treatment in the baseline strategy (SSOR) was US$2,746 in SA and US$2,270 in PH (Table 3). In SA, slow, moderate, or aggressive rollout of BPaL-based regimens would reduce the cost per successful treatment by 22%–28%, while in PH, this would reduce it by 13%–16%. In SA and PH, aggressive or moderate rollout would reduce the cost per successful treatment more than a slow rollout. To achieve the same number of successful treatments as baseline (SA: n = 28,251; PH: n = 54,268), total costs would decrease from US$78.3 million to US$56.7–61.1 million in SA (22%–28% reduction) and from US$123.1 million to US$103.8–107.8 million in PH (13%–16% reduction).

**Table 3.** Total expected number of people successfully treated, cost per successful treatment, and total cost to achieve the same number of successful treatments as baseline.

	Total number of people successfully treated (% change compared with baseline)	Cost per successful treatment (% change compared with baseline) in US$	Total costs required to achieve the same number of successful treatments as baseline in US$ (% change in total cost compared with baseline)
South Africa			
Baseline	28,521	2,746	7,83,26,127
Strategy 1	41,439 (45%)	1,987 (−28%)	56,657,234 (−28%)
Strategy 2	40,993 (44%)	2,005 (−27%)	57,181,585 (−27%)
Strategy 3	37,971 (33%)	2,141 (−22%)	61,064,739 (−22%)
Philippines			
Baseline	54,268	2,270	12,31,63,026
Strategy 1	64,539 (19%)	1,913 (−16%)	103,800,402 (−16%)
Strategy 2	64,240 (18%)	1,922 (−15%)	104,276,784 (−15%)
Strategy 3	62,160 (15%)	1,985 (−13%)	107,718,443 (−13%)

Rollout strategies: Baseline (all eligible patients initiated on SSOR); Strategy 1 = aggressive BPaLM/L rollout; Strategy 2 = moderate BPaLM/L rollout; Strategy 3 = slow BPaLM/L rollout.

### Budget impact analysis

Implementing BPaL-based regimens over 5 years would increase the treatment budget by 7%–8% in SA (from US$74 million with baseline to US$79–80 million, depending on the rollout strategy) and 6% in PH (from US$112 million with baseline to US$118–119 million). In SA, Strategies 1 (aggressive) and 2 (moderate) would initially increase the budget by 21% in 2023/2024, with subsequent annual increases of 6%–8%, reaching US$18.48 million in 2027/28, and a total 5-year budget of US$80 million (vs. US$74 million for baseline). Strategy 3 (slow) would have an annual increase of 5%–8%, reaching a US$79 million 5-year budget (Table 4).

**Table 4.** Total budget by fiscal year over 5 years and by rollout strategy, including percentage change relative to baseline.

	Total cost by budget year (million US$)					
	2023/24	2024/25	2025/26	2026/27	2027/28	5-year budget
South Africa						
Baseline	9.98	14.51	15.59	16.49	17.44	74.02
Strategy 1	12.07	15.70	16.52	17.48	18.48	80.25
% change over baseline	21	8	6	6	6	8
Strategy 2	12.07	15.40	16.58	17.59	18.48	80.12
% change over baseline	21	6	6	7	6	8
Strategy 3	10.76	15.74	16.53	17.59	18.37	78.98
% change over baseline	8	8	6	7	5	7
Philippines						
Baseline	10.08	17.30	21.83	27.55	34.77	111.54
Strategy 1	12.53	18.36	22.79	28.76	36.29	118.72
% change over baseline	24	6	4	4	4	6
Strategy 2	12.53	17.97	22.92	29.00	36.29	118.71
% change over baseline	24	4	5	5	4	6
Strategy 3	11.00	18.71	22.96	29.10	36.12	117.89
% change over baseline	9	8	5	6	4	6

Rollout strategies: Baseline (all eligible patients initiated on SSOR); Strategy 1 = aggressive BPaLM/L rollout; Strategy 2 = moderate BPaLM/L rollout; Strategy 3 = slow BPaLM/L rollout.

In PH, Strategies 1 and 2 would increase the budget by 24% in 2023/2024, followed by annual growth of 4%–6%, totalling US$119 million over 5 years (vs. US$112 million for baseline). Strategy 3 would initially increase by 9%, and then 4%–8% annually, totalling US$118 million over 5 years.

### Economic impact

Recent modelling studies estimate that between 2023/2024 and 2027/2028, BPaL treatment would avert 164,038 DALYs (healthy years saved) in SA and 214,585 DALYs in PH.^14^ We used this data and the GDP per capita to estimate the economic value of these health gains in SA and PH. Over 5 years, the economic benefit of BPaL-based regimens in SA would be US$987,918,855, and in PH, US$816,474,464. This far exceeds the initial budgetary increase required for the regimen rollout (Table 4).

### Sensitivity analysis

**Figure 2.**
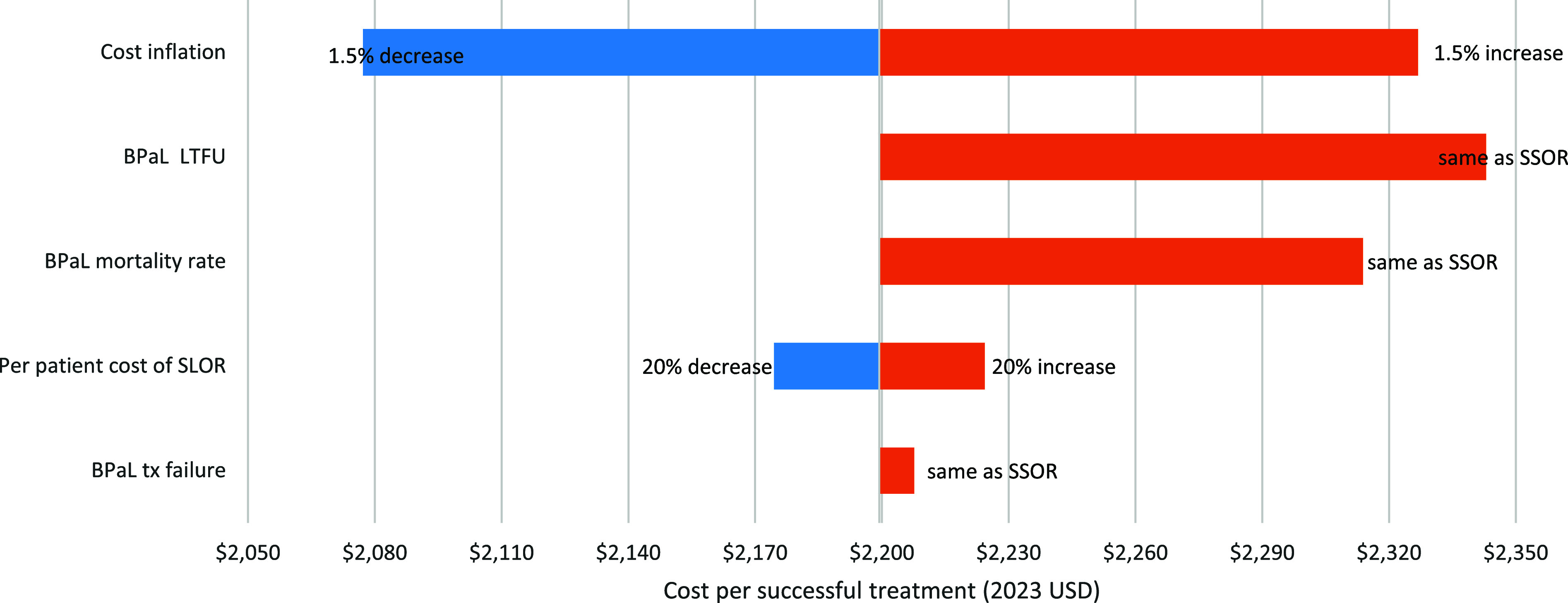
One-way sensitivity analysis of individual key input parameters. Cost per successful treatment as estimated in year 2027/28 for Strategy 2 in South Africa. SSOR = standard short oral regimen; SLOR = standard long oral regimen; LTFU = loss to follow-up; tx failure = treatment failure.

A 1.5% change in the cost inflation rate affected the cost per successful treatment by US$125 (Figure 2). Aligning BPaL-based regimen LTFU and mortality rates with SSOR increased the cost per successful treatment by US$144 and US$115, respectively. Aligning BPaL treatment failure with SSOR had minimal impact, increasing the cost per successful treatment by only US$8.

## DISCUSSION

BPaL-based regimens are projected to reduce the cost per successful treatment by 22%–28% in SA and 13%–16% in PH. Implementing them would require a 7%–8% budget increase in SA and 6% in PH. This increase, concentrated in Year 1, reflects higher upfront costs under Strategies 1 (aggressive) and 2 (moderate), which scale treatment to a larger proportion of eligible patients. As the regimen becomes established and outcomes improve, costs stabilise.^28^ SSOR’s poorer outcomes – high LTFU and mortality – lead to shorter treatment durations and lower resource use. In contrast, BPaL-based regimens are expected to improve treatment success by 26%, reducing LTFU and mortality. Better outcomes mean more patients complete treatment. This increases resource use (e.g., medications, monitoring visits, and laboratory tests), raising costs. However, the higher treatment success rates of BPaL-based regimens result in a lower cost per favourable outcome compared with SSOR, ultimately improving cost-effectiveness. Moreover, although the 5-year treatment budget is projected to rise, our analysis highlights the economic benefits of this investment. Improved treatment outcomes lead to significant societal gains, as each additional healthy year (DALY averted) enables individuals to work, earn income, support families, and contribute to the economy. Improved outcomes enhance TB control and positively impact a country’s economic well-being. Over 5 years, the economic benefit of BPaL-based regimens in SA would be US$987,918,855, and in PH, US$816,474,464. This far exceeds the initial budgetary increase required for regimen rollout.

Our analysis has several limitations. First, we derived rates of LTFU, treatment failure, and mortality for BPaLM/L and BPaL from BCAP/OR, where patients received rigorous monitoring and follow-up that may not reflect real-world settings – potentially overstating cost-reductions in terms of cost per person successfully treated. However, when assuming equivalent LTFU, treatment failure, and mortality rates as SSOR (Figure 2), the impact on cost per successful treatment was relatively minimal. Second, the duration of BPaL visits under routine programmatic conditions remains uncertain. For this analysis, we assumed visit lengths would be comparable to those in the BCAP/OR studies though this may be an overestimation. However, this assumption is consistently applied across all regimens and is not expected to affect the percentage differences between rollout strategies significantly. Third, based on PH PMDT projections, we assumed the number of patients treated in PH will increase over the next 5 years. However, our analysis did not incorporate the costs of identifying or bringing these patients into care, which would also apply to SSOR and would not affect the percentage change in costs we estimated. Fourth, as this was not a TB transmission modelling study, we did not account for reduced transmission associated with improved treatment rates – particularly given the uncertainty of the extent to which MDR/RR-TB patients contribute to onward transmission. If improved treatment rates decrease MDR/RR-TB incidence and recurrent TB, fewer individuals will need treatment, lowering the total budget. Therefore, our estimates likely overestimate the total budgetary requirements of BPaL-based regimens. Furthermore, the impact on the broader population transmission was not considered, potentially underestimating positive health gains. Fifth, while GDP per capita offers a standardised metric for economic analysis, it has limitations. It represents average economic output but does not account for income inequality. Moreover, this approach assumes that all healthy years directly contribute to income generation, potentially overstating the economic benefits of DALYs averted. However, using GDP per capita ensures consistency with previous studies, provides a recognised measure for comparing the economic benefits of health interventions, and helps translate health gains into tangible economic value. Finally, we did not account for any patients initiating an oral 18–21 month (SLOR) or longer individualised regimen (ITR) due to baseline resistance to pretomanid and/or bedaquiline and/or linezolid, as this is considered low or rare.^29^ For similar reasons, we did not track the costs of the SLOR/ITR. Additionally, when a patient switches due to short MDR/RR-TB regimen treatment failure, the episode is registered as a ‘treatment failure’.^3^ Therefore, we tracked patients only until a treatment outcome was assigned and not subsequent treatment episodes.

## CONCLUSIONS

BPaL-based regimens have a lower cost per successful treatment than SSOR. Implementing BPaL-based regimens may lead to 22%–28% cost savings in SA and 13%–16% in PH to achieve the same number of successful treatments as SSOR. Treating the same number of individuals, however, will increase the budget due to reduced LTFU and mortality (compared with SSOR) due to BPaL-based regimens’ effectiveness and simplicity. This increase accounts for full duration of care, including additional visits, medications, and lab tests to support a 26% improvement in treatment success while reducing LTFU, mortality, and treatment failure over 5 years. Investing in this approach will significantly enhance patient care and outcomes. Despite certain limitations, our model proved robust, with cost fluctuations of less than US$150 (7%) per successful treatment under key input variations, reinforcing the reliability of our findings.

## Supplementary Material


