# Supplementary material for: An economic analysis of BPaL for multidrug-resistant TB in South Africa and the Philippines
Source: IJTLD Open. 2025 Sep 10;2(9):535–41. doi: 10.5588/ijtldopen.25.0294 (PMC12435464; doi:10.5588/ijtldopen.25.0294)
Supplement: Supplementary file 1 [file ijtldopen25-0294_supplementarydata1.pdf]

Supplementary appendix

Supplementary Table 1. Summary of available literature on cost, cost savings, and outcomes of BPaL for rifampicin-resistant and drug-resistant TB.

Publication	Data source – costs	Data source – treatment outcomes	Treatment arms/groups comparison	Countries	Approach	Results	a. Cost per successful outcome (BPaL vs 9- or 18-m regimen<br>b. % cost saving
Evans et al., 2024a[3]<br><br>**RR-TB**	Bottom-up retrospective medical record review (patient resource-use) and top-down financial record review at the facilities	Retrospective medical record review (BCAP) and national electronic registry	BPaL vs 9-11-month SSOR or 18-21-month SLOR	South Africa	Bottom-up costing analysis consisting of a retrospective medical record review (patient resource-use) and top-down financial record review (fixed/shared costs such as overhead)	BPaL regimen was more cost-effective than SSOR and SLOR	a. US$4,948.7 per patient vs. US$4,905.6 for SSOR and US$8,919.9 for SLOR
Evans et al., 2024b[4]<br><br>**RR-TB**	Bottom-up/top-down (retrospective medical record review and facility-level financial record review); ingredients-based costing analysis (local facilities)	Retrospective medical record review and national public sector registry	BPaL vs 9-11-month SSOR or 18-21-month SLOR	the Philippine s	Bottom-up and top-down costing analysis	BPaL regimen was more cost-effective than SSOR and SLOR	a. US$1,994.5 per patient vs. US$3121.5 for SSOR and US$10,032.4 for SLOR<br>b. 36% less vs. SSOR; 80% less vs. SLOR
Auer et al., 2024[7]<br><br>**MDR-TB**<br>**Pre-XDR-TB**	Retrospective review of national records and estimates obtained from 'SLASH-TB' worksheet	Retrospective review of national records and estimates obtained from 'SLASH-TB' TB data worksheet	BPaL/BPaLM vs. SSOR/SLOR regimens	Pakistan[a]<br>South Africa<br>the Philippine s<br>Ukraine	-	BPaL/BPaLM regimens were cheaper than SSOR and SLOR	a. Pakistan: US$1,112 for BPaL/M per patient vs. US$2,271 for SSOR/SLOR;<br>The Philippines: US$1,685 for BPaL/M per patient vs. US$2,698 for SSOR/SLOR;<br>South Africa: US$1,437 for BPaL/M vs. US$3,268 for SSOR/SLOR;<br>Ukraine: US$2,379 for BPaL/M vs. US$7,969 for SSOR/SLOR (Ukraine)<br>b. Pakistan: 42%<br>The Philippines: 23%<br>South Africa: 38%<br>Ukraine: 51%

Publication	Data source – costs	Data source – treatment outcomes	Treatment arms/groups comparison	Countries	Approach	Results	a. Cost per successful outcome (BPaL vs 9- or 18-m regimen<br>b. % cost saving
James et al., 2024[8]<br><br>**RR-TB**	Published literature and other publicly available sources to parameterize a mathematical model estimating lifetime costs	Publicly available data/published literature to parameterize a mathematical model (Markov microsimulation) estimating long-term health outcomes (QALYs)	BPaLM (6-month) and other regimens vs 9- or 18-month SOC treatment<br><br>• BPaLM→BPaLC[b]<br>• BPaLM→BPaLC[c]<br>• BPaLM→BPaLC[d]<br>• BPaLM→BPaL[e]<br>• BPaLM→BPaL[f]<br>• BPaLM→BPaL[g]<br>• SOC[h]<br>• SOC[i]	Moldova	Markov modelling. Top-down estimates for public sector based on published literature	6 months of BPaLM was more cost-effective compared to the SOC	a. Discounted total cost: BPaLM→BPaLC[b] US$8,153, BPaLM→BPaLC[c] US$8,167, BPaLM→BPaLC[d] US$8,398, BPaLM→BPaL[e] US$8,723, BPaLM→BPaL[f] US$8,750, BPaLM→BPaL[g] US$9,039 vs. SOC[h] US$ 11,534 or SOC[i] US$ 11,816<br>b. US$3,366 difference between BPaLM→BPaLC[c] and SOC[h]
Sweeney et al., 2022[9]<br><br>**RR-TB**	Treatment costs were estimated from a providers' perspective using a combination of country MDR treatment guidelines, previously published estimates of prices and quantities of different services, and expert opinion	TB-PRACTECAL trial - outcomes for SOC regimens in the Markov model were based on national treatment outcomes from the 2019 treatment cohort in each country, as reported to WHO	BPaL, BPaLC, BPaLM vs SOC regimen mix	the Philippines<br>South Africa<br>India<br>Georgia	Markov trial cohort modelling	BPaL was the more cost-effective regimen compared to the SOC	a. The Philippines: US$1,078 (BPaL), US$1,174 (BPaLC), US$1,124 (BPaLM) vs. US$1,329 for SOC<br>South Africa:<br>US$3,344 (BPaL), US$3,470 BPaLC), US$3,520 (BPaLM) vs. $4,517 for SOC<br>India:<br>US$867 (BPaL), US$952 BPaLC), US$899 (BPaLM) vs. US$978 for SOC<br>Georgia:<br>US$3,228 (BPaL), US$3,327 BPaLC), US$3,307 (BPaLM) vs. US$4,211 for SOC
Fekadu et al., 2024[10]<br>**HDR-TB**	-	A lifelong decision-analytic model was constructed to simulate potential treatment outcomes	BPaL vs. B-L	US	Lifelong decision-analytic model	BPaL therapy is likely the cost-effective option for HDR-TB treatment	a. -<br>b. saved costs by US$60,433

Publication	Data source – costs	Data source – treatment outcomes	Treatment arms/groups comparison	Countries	Approach	Results	a. Cost per successful outcome (BPaL vs 9- or 18-m regimen b. % cost saving
Gomez et al., 2021[5]<br><br>**XDR-TB**	Cost estimates from the literature and local consultations and guidelines were used to build disaggregated unit costs for the intervention and comparator (i.e., drugs, visits, tests). Data were obtained from the Global Health Costing Consortium database and from VALUE TB (a multi-country TB costing study funded by the Bill and Melinda Gates Foundation)	Markov cohort model parameterised with a combination of publicly available and aggregated health outcome data	BPaL vs. 18-month SOC regimens	South Africa Georgia the Philippines	Markov cohort modelling	BPaL estimated to be cost-saving to the local TB programmes in varied programmatic settings	a. South Africa: US$1,859,461 vs. US$5,206,829 for SOC (XDR-TB cohort) Georgia: US$83,775 vs. US$282,680 for SOC (XDR-TB cohort) The Philippines: US$26,357 vs. US$84,327 for SOC (XDR-TB cohort)<br>b. -
Mulder et al., 2022[6]<br><br>**XDR-TB**	Landscape analysis followed by bottom-up costing method for health services costs; Top-down analysis from reviewed literature (including estimates from World Health Organization Choosing Interventions that are Cost-Effective (WHO-CHOICE)), price catalogues, financial records service utilisation statistics (online supplemental Cost Analysis)	Markov modelling	BPaL vs. conventional regimens including bedaquiline, clofazimine and other drugs (varying by country)	Indonesia, Kyrgyzstan, Nigeria	Bottom-up and top-down cost methods, Markov modelling	BPaL regimen can be a highly cost-saving regimen compared with conventional XDR-TB regimens	a. Indonesia: US$7,142 per patient completing treatment vs US$16,732 Kyrgyzstan: US$4,782 per patient completing treatment vs. US$21,714 Nigeria: US$7152 per patient completing treatment vs. US$22,021<br>b. 57%, 78%, 68% less vs. conventional regimens, respectively

BPaLC bedaquiline-pretomanid-linezolid-clofazimine; *BPaL* bedaquiline-pretomanid-linezolid; *BPaLM* bedaquiline-pretomanid-linezolid-moxifloxacin; *BPaLL* bedaquiline-pretomanid-linezolid-levofloxacin; *BCAP*, clinical access programme; *DST* drug susceptibility test; *MDR-TB* multidrug-resistant tuberculosis; *SLASH-TB* Savings from Leveraging & Adopting Shorter & Highly Effective TB Treatments; *SLOR* standard long oral regimen; *SOC*, standard of care; *SSOR* standard short oral regimen; *QALY* quality-adjusted life years; *XDR-TB* extensively drug-resistant tuberculosis; *HDR* high drug-resistant

[a]The Philippines is already using BPaL, and SLASH-TB uses the actual treatment outcomes for BPaL and TB-PRACTECAL results for BPaLM to calculate the effectiveness. Zenix trial results were also used for BPaL and interim published results for BPaLM in TB-PRACTECAL, both showing a lower treatment success rate.

[b] Second line DST at treatment initiation = no; routine frequency of subsequent second line DST = every 4 months. [c] Second line DST at treatment initiation = yes; routine frequency of subsequent second line DST = every 4 months. [d] Second line DST at treatment initiation = yes; routine frequency of subsequent second line DST = every 1 month. [e] Second line DST at treatment initiation = no; routine frequency of subsequent second line DST = every 4 months. [f] Second line DST at treatment initiation = no; routine frequency of subsequent second line DST = every 4 months month. [g] Second line DST at treatment initiation = yes; routine frequency of subsequent second line DST = every 1 month. [h] Second line DST at treatment initiation = yes; routine frequency of subsequent second line DST = every 4 months. [i] Second line DST at treatment initiation = yes; routine frequency of subsequent second line DST = every 1 month.

Text S1. Transition probabilities and cost data sources

The BPaL clinical access program (BCAP) started in 2020 and planned to enrol up to 400 participants ⩾14 years in the single-arm open-label intervention study for treatment with a three-drug, 24-week, all-oral regimen of bedaquiline, pretomanid and linezolid (BPaL) regimen. BCAP took place at multiple sites, including Durban and Johannesburg, South Africa. Based on the favourable outcomes from clinical trials[17-19] and the initial BCAP results, we assumed that no BPaL patients failed treatment or were LTFU. Additionally, we made the following assumptions; transition probabilities for BPaLM/L would replicate those observed for BPaL; and among those who initially initiated initially on BPaLM/L, 20% would transition to BPaL due to fluoroquinolone resistance,[20] while 2% would switch from BPaL to a longer oral regimen (SLOR) due to treatment failure or intolerence.[19] This transition was assumed to occur within the first month of treatment commencement.
Supplementary Table S3 outlines the monthly transition probabilities between disease states, while Table 1 presents the cumulative transition rates per regimen and country throughout the treatment duration.

The operational research (OR) was conducted between 2021 and 2023 and compared the cost and cost-effectiveness of BPaL to the 9-11 month short and the 18-24 month long oral regimen under programmatic conditions.[3, 4] The BCAP/OR costing studies were conducted according to the methods outlined in the WHO costing guidelines for TB interventions.[21]

Table S3. Assumed transition rates between disease states.

**Disease state and Month**	SSOR*		BPaLM/L^		BPaL^		SLOR*	
	South Africa	Philippines	South Africa	Philippines	South Africa	Philippines	South Africa	Philippines
Loss to follow up								
1	1.21%	1.17%	0.00%	0.00%	0.00%	0.00%	0.90%	0.84%
2	1.29%	1.46%	0.00%	0.00%	0.00%	0.00%	0.96%	0.77%
3	1.55%	1.42%	0.00%	0.00%	0.00%	0.00%	1.14%	0.65%
4	1.82%	1.00%	0.00%	0.00%	0.00%	0.00%	1.49%	0.59%
5	1.99%	0.73%	0.00%	0.00%	0.00%	0.00%	1.46%	0.43%
6	2.00%	0.63%	0.00%	0.00%	0.00%	0.00%	2.00%	0.35%
7	1.93%	0.62%	0.00%	0.00%	0.00%	0.00%	2.34%	0.37%
8	1.83%	0.47%	0.00%	0.00%	0.00%	0.00%	2.05%	0.19%
9-12	4.35%	0.46%	0.00%	0.00%	0.00%	0.00%	6.63%	1.43%
13 and above	11.50%	1.44%	0.00%	0.00%	0.00%	0.00%	10.90%	2.06%
Treatment failure								
1	0.07%	0.14%	0.00%	0.00%	0.00%	0.00%	0.25%	0.49%
2	0.10%	0.00%	0.00%	0.00%	0.00%	0.00%	0.11%	0.46%
3	0.01%	0.07%	0.00%	0.00%	0.00%	0.00%	0.16%	0.33%
4	0.06%	0.07%	0.00%	0.00%	0.00%	0.00%	0.11%	0.00%
5	0.09%	0.02%	0.00%	0.00%	0.00%	0.00%	0.19%	0.09%
6	0.13%	0.12%	0.00%	0.00%	0.00%	0.00%	0.23%	0.18%
7	0.24%	0.18%	0.00%	0.00%	0.00%	0.00%	0.36%	0.18%
8	0.29%	0.24%	0.00%	0.00%	0.00%	0.00%	0.33%	0.37%
9-12	0.88%	0.33%	0.00%	0.00%	0.00%	0.00%	1.62%	1.71%
13 and above	2.38%	2.39%	0.00%	0.00%	0.00%	0.00%	2.99%	2.16%
Death								
1	6.94%	2.92%	2.56%	2.56%	2.56%	2.56%	4.76%	7.60%
2	3.21%	1.41%	0.00%	0.00%	0.00%	0.00%	2.74%	4.23%
3	1.83%	1.11%	0.00%	0.00%	0.00%	0.00%	2.19%	2.28%
4	1.24%	0.87%	0.00%	0.00%	0.00%	0.00%	1.80%	2.44%
5	1.20%	0.71%	0.00%	0.00%	0.00%	0.00%	1.31%	1.30%
6	1.01%	0.51%	0.00%	0.00%	0.00%	0.00%	1.15%	1.59%
7	0.86%	0.57%	0.00%	0.00%	0.00%	0.00%	0.91%	1.01%
8	0.93%	0.47%	0.00%	0.00%	0.00%	0.00%	0.85%	0.84%
9-12	1.57%	0.26%	0.00%	0.00%	0.00%	0.00%	3.88%	2.57%
13 and above	4.49%	0.72%	0.00%	0.00%	0.00%	0.00%	5.81%	2.88%

* from Electronic Drug-Resistant Tuberculosis Register (EDRWeb); ^ South African BPaL clinical access program (BCAP)
